# Plasma triglyceride and high density lipoprotein cholesterol are poor surrogate markers of pro-atherogenic chylomicron remnant homeostasis in subjects with the metabolic syndrome

**DOI:** 10.1186/s12944-016-0330-4

**Published:** 2016-09-29

**Authors:** Deasy Irawati, John C. L. Mamo, Satvinder S. Dhaliwal, Mario J. Soares, Karin M. Slivkoff-Clark, Anthony P. James

**Affiliations:** 1 School of Public Health, Faculty of Health Sciences, Curtin University, Bentley, GPO Box U1987, Perth, WA 6845 Australia; 2 Curtin Health Innovation Research Institute, Curtin University, Perth, WA Australia; 3 Faculty of Medicine, Mataram University, West Nusa Tenggara, Indonesia

**Keywords:** Metabolic syndrome, Chylomicron, Apolipoprotein B-48

## Abstract

**Background:**

Subjects with metabolic syndrome (MetS) exhibit impaired lipoprotein metabolism and have an increased risk of cardiovascular disease. Although the risk is attributed primarily to the risk associated with individual components, it is also likely affected by other associated metabolic defects. Remnants of postprandial lipoproteins show potent atherogenicity in cell and animal models of insulin resistance and in pre-diabetic subjects with postprandial dyslipidemia. However, few studies have considered regulation of chylomicron remnant homeostasis in MetS per se. This study measured the plasma concentration in Caucasian men and women of small dense chylomicrons following fasting and explored associations with metabolic and anthropometric measures.

**Methods:**

A total of 215 Australian Caucasian participants (median age 62 years) were investigated. Of them, 40 participants were classified as having MetS. Apolipoprotein (apo) B-48, an exclusive marker of chylomicrons, metabolic markers and anthropometric measures were determined following an overnight fast.

**Results:**

The fasting apo B-48 concentration was 40 % higher in subjects with MetS than those without MetS. In all subjects, triglyceride (*r* = 0.445, *P* < 0.0005), non-HDL cholesterol (*r* = 0.28, *P* < 0.0005) and HDL cholesterol concentration (*r* = −0.272, *P* < 0.0005) were weakly associated with apo B-48 concentration. In subjects with MetS, the association of apo B-48 with triglyceride and non-HDL cholesterol was enhanced, but neither were robust markers of elevated apo B-48 in MetS (*r* = 0.618 and *r* = 0.595 respectively). There was no association between apo B-48 and HDL cholesterol in subjects with MetS.

**Conclusion:**

This study demonstrates a substantial accumulation of pro-atherogenic remnants in subjects with MetS. We have shown that in a Caucasian cohort, the fasting plasma concentration of triglyceride or HDL/non-HDL cholesterol serves as poor surrogate markers of atherogenic chylomicron remnants. These findings suggest that subjects with MetS exhibit a chronic defect in chylomicron metabolism that is likely to contribute to their increased CV risk.

## Background

It is recognised that patients with metabolic syndrome (MetS) are at risk developing CVD [1–3]. Increased plasma triglyceride and decreased HDL cholesterol are important components of MetS syndrome, however subjects with MetS are otherwise typically normocholesterolemic.

A substantial body of evidence supports the contention that hydrolysed remnants of postprandial chylomicrons are highly atherogenic [4, 5]. Apo B-48 concentration, a unique marker of chylomicron particles, is associated with atherosclerosis and coronary artery disease [4, 6, 7]. The presence of apo B-48 containing particles in rabbit and human atherosclerotic tissue [8–10] suggests that post-hydrolyzed chylomicron remnants are an important risk for development of atheroma. It was previously thought that chylomicrons are synthesized and released by the intestine only after dietary fat ingestion and that they are primarily found in lipid-rich lipoprotein fractions. However, we and others have reported the presence of chylomicron particles in more dense, lipid-poor lipoprotein fractions in the absence of dietary fat availability [11–13]. Small sized (remnant) chylomicron particles are able to penetrate the arterial wall and get trapped within the subendothelial space [14, 15]. This can cause lipid accumulation in macrophages, a hallmark of early atherogenesis, without prior chylomicron remnant oxidative modification [16, 17].

Subjects with MetS have been found to have greater fasting concentrations of apo B-48 [18–20]. However these observations have been made in a Japanese population and hence have been grouped using the Japanese Metabolic Syndrome criteria [7, 18, 20–22]. It is therefore important to examine the association between apo B-48 and metabolic parameters in other populations.

The metabolic aberrations leading to an accumulation of chylomicron remnants in MetS are unclear, but studies in animal models and insulin resistant subjects suggest exaggerated rates of constituent biosynthesis as a consequence of intestinal hypertrophy [23]; decreased lipolysis because of reduced expression of endothelial lipoprotein lipase [24] and thereafter, lower rates of receptor mediated endocytosis post-hydrolysis contribute that collectively results in increased vascular exposure.

Plasma triglyceride is commonly utilized as a marker of postprandial lipaemia and indeed fasting levels have been reported to correlate with fasting apo B-48 concentration [18, 21, 22]. However, plasma triglyceride principally reflects the lipolytic cascade and is by extension less indicative of accumulation of post-hydrolyzed atherogenic remnants. Greater plasma abundance of triglyceride is commonly associated with reduced plasma concentration of HDL cholesterol. The latter reflects principally, decreased shedding of surface phospholipids and cholesterol from triglyceride rich lipoproteins during lipolysis and genesis of nascent discoidal HDL lipoproteins. In MetS, heightened plasma triglyceride and/or low HDL cholesterol may be associated with an accumulation in plasma of atherogenic chylomicron remnants, however neither measure are equivocal surrogate markers of remnant homeostasis per se.

Apo B-48 is produced exclusively by absorptive cells of the small intestine and is an obligatory moiety of nascent chylomicrons. In normolipidemic subjects with coronary artery disease and in subjects with familial hypercholesterolemia, apo B-48 is markedly elevated despite otherwise normal levels of plasma triglyceride [25, 26]. The plasma abundance of small dense chylomicrons in Caucasian subjects with MetS has not been previously reported. Moreover, altered chylomicron homeostasis in MetS may be associated with one or more of the MetS components, however this has not been directly considered. This study reports on associations of fasting apo B-48 with metabolic and anthropometric measures in Caucasian men and women.

## Methods

### Subjects

Two hundred and fifteen Australians with European ancestry were sampled randomly from a larger cross sectional study investigating the relationship between serum vitamin D, bioactive calcium, parathyroid hormone homeostasis and cognitive performance conducted at our institute in 2011 [27]. An additional exclusion criteria was used for the present study to exclude subjects on lipid lowering medication. MetS criteria was based on the new harmonising criteria of Alberti et al. [28]. All participants have given written informed consent prior participating in the study. The study has been approved by the Curtin University Human Research Ethics Committee (HR97/2011) and was performed in accordance to Declaration of Helsinki.

### Anthropometric measurements

After having fasted for at least 8 h, prior to fasting blood collection, a range of anthropometric measurements were performed. Body weight was measured after voiding, with participants wearing limited clothing on a digital balance and recorded to the nearest 100 g. Standing height was measured using a portable stadiometer fixed to the wall, without shoes and recorded to the nearest 0.1 cm. Waist circumference and ratio was measured and calculated as described by Norton and Olds [29]. Sagittal abdominal diameter (SAD) was measured by using an abdominal calliper while the participants were in a standing position. The SAD was measured as the distance between the largest point of abdomen and the back at the end of normal expiration. Blood pressure will be assessed using a semi-automatic (arm cuff) blood pressure monitor (Omron, Vernon Hills, IL) in supine position. All measurements were done by a certified anthropometrist.

### Lipid, insulin and glucose assays

Fasting serum samples were collected by low-speed centrifugation of serum separator Vacutainer™ tubes (Becton Dickinson, Franklin Lakes, NJ, USA) that were left to clot for 30 min. All samples were then either analysed immediately or stored at −80 °C prior to analysis. Fasting serum triglyceride, cholesterol and glucose were determined by Pathwest Laboratories using their routine automated procedures on an Architect c1600 analyser. Serum triglyceride, total cholesterol, HDL cholesterol and glucose concentration were measured by using specific enzyme-based colorimetric reagents (Abbott Diagnostics, Abbott Laboratories, Abbott Park, USA; CV <2 %). LDL cholesterol was estimated by using a modified version of Friedewald formula [30]. Non-HDL cholesterol concentration was calculated as the difference between serum total cholesterol and HDL cholesterol concentration. Insulin level was determined by using the Mercodia insulin ELISA kit (Mercodia AB, Uppsala, Sweden) according to the manufacturer’s instructions. Insulin resistance was assessed by calculation of HOMA-IR score [31].

### Apo B-48 determination

Chylomicron concentration was measured by determining apo B-48 concentration. Apo B-48 is the major structural protein for chylomicron assembly and it is not interchangeable [32]. Apo B-48 concentration was measured by using a commercial sandwich ELISA method using a monoclonal antibody raised against the C-terminal region of apo B-48 (Shibayagi Human apo B-48 ELISA Kit, Ishihara, Shibukawa, Japan) according to the manufacturer’s instructions. The details of the validation method refers to Kinoshita et al. [33].

### Statistical analysis

All data were analysed using SPSS version 21 (SPSS Inc., Chicago, USA). The results are all presented in the tables as mean values ± standard error (SE) for normally distributed data and median (25^th^ and 75^th^ interquartile range) if the data was skewed. Data that were not normally distributed were natural log transformed when necessary. Statistical significance was assessed using Independent-samples *T* test, Mann-Whitney test (between 2 groups) and ANOVA (3 groups or more). Post-hoc comparison between each group was assessed using Bonferroni test. To assess the significant difference in apo B-48 between the MetS and no-MetS group, GLM univariate were used. Spearman correlations were computed to assess the association between parameters. The strength of association between apo B-48 and a range of anthropometric and metabolic determinants adjusted for age and gender were calculated by using Z score derived regression coefficient between apo B-48 and other variables. Regression coefficient denotes the standard deviation change in apoB-48, as a result of 1 standard deviation change in the variable. A *p* value < 0.05 was considered significant.

## Results

### Subject characteristics

The subject characteristics are shown in Table 1. The median age of the whole subjects was 62 years. Subjects with MetS were older and had higher anthropometric measures related to overweight (BMI, waist circumference, WHR and SAD) and were more likely to be dyslipidemic (high triglyceride and/or low HDL cholesterol). Subjects with MetS exhibited a higher non-HDL cholesterol concentration than those without MetS, but no significant differences in total cholesterol or LDL cholesterol concentration were observed in the MetS group. They were also more insulin resistant than those without MetS group.

**Table 1:** Subject characteristics

Characteristics	Total (*n* = 215)	No-MetS (*n* = 175)	MetS (*n* = 40)
Gender (Male/Female)	68/147	46/129	22/18
Age (years)	62 (56–69)	62 (56–68)	66 (59–71)*
BMI (kg/m^2^)	26.3 ± 0.3	25.5 ± 0.3	29.9 ± 0.6**
Waist circumference (cm)	87.1 ± 0.9	84.1 ± 1	100.3 ± 1.4**
WHR	0.83 ± 0.01	0.81 ± 0.01	0.92 ± 0.01**
SAD (cm)	24.7 ± 0.3	23.6 ± 0.3	29.3 ± 0.5**
Systolic BP (mmHg)	141 ± 1	139 ± 2	150 ± 3**
Diastolic BP (mmHg)	83 ± 1	82 ± 1	85 ± 2
Triglyceride (mmol/L)	1.1 ± 0.03	1 ± 0.03	1.7 ± 0.1**
Total chol (mmol/L)	5.4 ± 0.1	5.4 ± 0.1	5.5 ± 0.2
LDL chol (mmol/L)	3.3 ± 0.1	3.3 ± 0.1	3.5 ± 0.1
HDL chol (mmol/L)	1.5 ± 0.03	1.6 ± 0.03	1.2 ± 0.03**
Non-HDL chol (mmol/L)	3.9 ± 0.1	3.8 ± 0.1	4.3 ± 0.2**
Glucose (mmol/L)	5.1 (4.8–5.4)	5.1 (4.8–5.3)	5.6 (5.3–5.9)**
Insulin (mIU/L)	6 ± 0.2	5.4 ± 0.2	8.8 ± 0.5**
HOMA-IR	1.4 ± 0.1	1.2 ± 0.04	2.2 ± 0.1**
CRP (μg/mL)	2.9 ± 0.2	2.6 ± 0.2	4.3 ± 0.4**
ApoB-48 (μg/mL)	4.8 (3.4–7.9)	4.6 (3.3–7.5)	6.8 (3.9–11.4)**
Metabolic parameters		Number of subjects	
BMI ≥ 25 kg/m2	129 (60 %)	91 (52 %)	38 (95 %)
Large waist	104 (48 %)	68 (39 %)	36 (90 %)
Triglyceride ≥ 1.7 mmol/L	31 (14 %)	6 (3 %)	25 (63 %)
Low HDL chol	30 (14 %)	10 (5 %)	20 (50 %)
Hypertension	147 (69 %)	111 (63 %)	36 (92 %)
Fasting glucose ≥ 5.6 mmol/L	32 (15 %)	11 (6 %)	21 (53 %)

Mean ± SE for normally distributed variables or median (25^th^ and 75^th^ interquartile range) for non-normally distributed variables; *p < 0.05, **p < 0.01; MetS compared to no-MetS group. *P* values between groups were based on independent *t*-test and Mann-Whitney test for data that is normally and non-normally distributed data respectively

### Apo B-48, anthropometric and lipid parameters in MetS

The median serum apo B-48 concentration for the whole group was 4.8 μg/mL (3.4–7.9) (Table 1). Subjects with MetS exhibited a 40 % greater apo B-48 concentration than those without MetS (Table 2; *P* < 0.01). The difference between groups remained significant following adjustment for age and gender (Table 2). In subjects not classified as having MetS, the concentration of apo B-48 did not appear to be affected by the presence of 0, 1 or 2 MetS components.

**Table 2:** Concentration of apo B-48 according to MetS and in different cumulative number of MetS components

Presence of MetS	N	Geometric mean (95 % CI) (μg/mL)
No-Mets	175	4.7 (4.3–5.2)
MetS	40	6.6 (5.4–8.0)
		*P* = 0.001*
MetS components		
0	37	4.7 (3.8–5.9)^a^
1	69	4.7 (4–5.6)^a^
2	69	4.7 (4.1–5.3)^a^
≥3	40	6.6 (5.4–8)^b^
		*P* = 0.004*

*p value after adjusting for age and gender

Different letters (a and b) indicate significant differences

The association between fasting apo B-48 and the range of outcome measures was examined in all subjects and also after grouping based on the presence of MetS. When examined on the whole group, apo B-48 concentration was found to correlate with triglyceride (*r* = 0.445, *p* < 0.0005), non-HDL (*r* = 0.28, *p* < 0.0005) and HDL cholesterol concentrations (*r* = −0.272, *p* < 0.0005) (Table 3). Weak correlations were observed between apo B-48 concentration both LDL cholesterol (*r* = 0.191, *p* = 0.005) and total cholesterol (*r* = 0.14, *p* = 0.042). Apo B-48 concentration was also weakly associated with waist circumference and WHR (*r* = 0.162, *P* = 0.017 and *r* = 0.178, *P* = 0.009). However no association was observed between apo B-48 and either insulin concentration, or HOMA-IR level in all subjects.

**Table 3:** Non-parametric correlations between anthropometric and lipid/metabolic determinants with Apo B-48 (without adjustment)

Variables	Correlation coefficient (*p* value)		
	Total (n = 215)	No MetS (n = 175)	MetS (N = 40)
Triglyceride^a^	**0.445 (** ***p*** **< 0.0005)**	**0.362 (** ***p*** **< 0.0005)**	**0.618 (** ***p*** **< 0.0005)**
HDL cholesterol^a^	**−0.272 (** ***p*** **< 0.0005)**	**−0.278 (** ***p*** **< 0.0005)**	0.254 (*p* = 0.113)
Age	−**0.230 (** ***p*** **= 0.001)**	**−0.262 (** ***p*** **< 0.0005)**	**−0.32 (** ***p*** **= 0.044)**
Non-HDL cholesterol	**0.28 (** ***p*** **< 0.0005)**	**0.157 (** ***p*** **= 0.038)**	**0.595 (** ***p*** **< 0.0005)**
WHR	**0.178 (** ***p*** **= 0.009)**	0.147 (*p* = 0.053)	−0.26 (*p* = 0.105)
Waist circumference^a^	**0.162 (** ***p*** **= 0.017)**	0.123 (*p* = 0.106)	**−0.317 (** ***p*** **= 0.046)**
Systolic^a^	−0.053 (*p* = 0.441)	−0.106 (*p* = 0.163)	−0.026 (*p* = 0.875)
LDL cholesterol	**0.191 (** ***p*** **= 0.005)**	0.094 (*p* = 0.214)	**0.533 (** ***p*** **< 0.0005)**
BMI	0.105 (*p* = 0.123)	0.078 (*p* = 0.308)	−0.27 (*p* = 0.092)
CRP	−0.006 (*p* = 0.929)	−0.073 (*p* = 0.335)	−0.005 (*p* = 0.977)
Insulin	0.113 (*p* = 0.1)	0.069 (*p* = 0.365)	−0.158 (*p* = 0.331)
Glucose^a^	−0.001 (*p* = 0.984)	−0.067 (*p* = 0.381)	−0.222 (*p* = 0.168)
SAD	0.118 (*p* = 0.089)	0.06 (*p* = 0.439)	−0.193 (*p* = 0.247)
HOMA-IR	0.108 (*p* = 0.113)	0.058 (*p* = 0.446)	−0.201 (*p* = 0.214)
Diastolic^a^	0.024 (*p* = 0.729)	0.023 (*p* = 0.761)	−0.066 (*p* = 0.691)
Cholesterol	**0.139 (** ***p*** **= 0.042)**	0.021 (*p* = 0.78)	**0.576 (** ***p*** **< 0.0005)**

The variables were presented in order based on the strength of association (strong to weak) in the no MetS group. Cells represent Spearman’s Rank correlation (*p*-values). Significant correlations are in bold font

^a^Variable use to determine the presence of MetS

When grouped based on MetS status, subjects with MetS exhibited an enhanced association of apo B-48 with triglyceride, total- (*r* = 0.618, *p* < 0.0005), non-HDL (*r* = 0.595, *p* < 0.0005) and LDL cholesterol (*r* = 0.576, *p* < 0.0005) than those without MetS (Table 2). However a negative association with HDL cholesterol concentration and apo B-48 was only observed in subjects who did not exhibit MetS. Waist circumference was inversely correlated with apo B-48 in the MetS group.

Since apo B-48 concentration was associated with age, we then assessed the association with correcting for age and gender (Table 4). We observed a minor enhancement in the association between apo B-48 and triglyceride concentration in both groups. The magnitude of association between apo B-48 and several cholesterol profiles (total cholesterol, non-HDL and LDL cholesterol) was greater in the MetS group compared to the no MetS group. However the contribution of those variables on the variability of apo B-48 was less than 40 %. We also observed that in the MetS group, apo B-48 was inversely associated with BMI and insulin. To assess the contribution of the lipid variables strongly associated with apo B-48 concentration, we combined the triglyceride and non-HDL cholesterol in a model adjusted for age and gender. The contribution of these variables to apo B-48 variability was 23.3 % (*R*^2^ = 0.233) in subjects without MetS and 45.1 % (*R*^2^ = 0.451) in those with MetS.

**Table 4:** The association between Z score of apo B-48 and anthropometric and lipid/metabolic determinants, after adjustment for age and gender

Variables	Regression coefficient based on Z-scores	
	No MetS	MetS
Triglyceride^a^	**0.534 (** ***p*** **< 0.0005,** ***R*** ^**2**^ **= 0.233)**	**0.634 (** ***p*** **= 0.001,** ***R*** ^**2**^ **= 0.436)**
Non-HDL cholesterol	**0.204 (** ***p*** **= 0.007,** ***R*** ^**2**^ **= 0.135)**	**0.546 (** ***p*** **= 0.008,** ***R*** ^**2**^ **= 0.378)**
Glucose^a^	−0.152 (*p* = 0.208, *R* ^2^ = 0.105)	−0.057 (*p* = 0.668, *R* ^2^ = 0.245)
LDL cholesterol	**0.149 (** ***p*** **= 0.047,** ***R*** ^**2**^ **= 0.118)**	**0.458 (** ***p*** **= 0.039,** ***R*** ^**2**^ **= 0.327)**
Cholesterol	0.144 (*p* = 0.059, *R* ^2^ = 0.116)	**0.547 (** ***p*** **= 0.009,** ***R*** ^**2**^ **= 0.374)**
HDL cholesterol^a^	−0.132 (*p* = 0.082, *R* ^2^ = 0.113)	0.154 (*p* = 0.693, *R* ^2^ = 0.244)
Waist circumference^a^	0.077 (*p* = 0.336, *R* ^2^ = 0.102)	−0.453 (*p* = 0.092, *R* ^2^ = 0.299)
WHR	0.073 (*p* = 0.453, *R* ^2^ = 0.1)	0.077 (*p* = 0.791, *R* ^2^ = 0.242)
Systolic^a^	−0.064 (*p* = 0.371, *R* ^2^ = 0.101)	0.06 (*p* = 0.782, *R* ^2^ = 0.278)
SAD	0.058 (*p* = 0.468, *R* ^2^ = 0.097)	−0.420 (*p* = 0.092, *R* ^2^ = 0.317)
BMI	0.053 (*p* = 0.443, *R* ^2^ = 0.1)	**−0.603 (** ***p*** **= 0.002,** ***R*** ^**2**^ **= 0.422)**
CRP	−0.039 (*p* = 0.57, *R* ^2^ = 0.099)	−0.153 (*p* = 0.375, *R* ^2^ = 0.257)
Insulin	0.014 (*p* = 0.896, *R* ^2^ = 0.097)	**−0.428 (** ***p*** **= 0.049,** ***R*** ^**2**^ **= 0.319)**
Diastolic^a^	0.013 (*p* = 0.846, *R* ^2^ = 0.097)	−0.165 (*p* = 0.377, *R* ^2^ = 0.293)
HOMA-IR	−0.005 (*p* = 0.972, *R* ^2^ = 0.097)	−0.429 (*p* = 0.069, *R* ^2^ = 0.308)

Regression coefficient represents the standard deviation change in apoB-48, as a result of 1 standard deviation change in the variable. The variables were presented in orders based on the strength of association (strong to weak) in the no MetS group. Cells represent: slope estimate (*p*-value for estimate, R^2^ for model after adjustment). Significant associations are in bold font

^a^Variable use to determine the presence of MetS

## Discussion

In this study, we have examined the importance of chylomicron remnants, as measured by apo B-48, in subjects with and without MetS. We report that subjects with MetS had approximately 40 % higher fasting concentration of apo B-48 than those without MetS. The association between the fasting concentration of chylomicron remnants and a range of anthropometric and lipid measures was also examined in subjects with and without MetS. In the whole group apo B-48 concentration correlated with fasting triglyceride concentration and cholesterol profile. In subjects with MetS these correlations increased in strength but as only 45 % of the variability in apo B-48 was predicted by the fasting concentration of triglyceride and non-HDL cholesterol, we conclude that these are not robust markers of chylomicron remnant homeostasis. This conclusion suggests that there are a significant proportion of subjects with MetS that have high apo B-48, but normal triglyceride and cholesterol profile; and hence the importance of apo B-48 determination in subjects with MetS.

Our observation that subjects with MetS exhibit a 40 % higher fasting concentration of apo B-48 compared to those without suggests an impairment in chylomicron metabolism in these subjects. Our findings confirm and extend those previously reported in a Japanese population where it was reported that subjects with MetS had on average a 34 % or 42 % higher fasting apo B-48 concentration (in males and females respectively) compared to control subjects without MetS [18]. Interestingly we also observed that elevated apo B-48 concentration was only observed in subjects who exhibited three or greater MetS components (and hence exhibited MetS) suggesting that impaired chylomicron metabolism is reliant on the altered phenotype of MetS rather than simply being a consequence of its individual parameters. Chylomicron metabolism may be impaired in subjects with MetS at the level of production, lipolysis and/or clearance. Increased production of apo B-48 particles via elevated availability of substrate in enterocytes (i.e. due to increased expression of NPC1L1), increased MTP activity, decreased LPL activity and reduced LDL receptor and LDL receptor protein activity have been proposed as a possible mechanism of the altered chylomicron metabolism [34, 35]. In subjects with MetS, hepatic overproduction of VLDL has been reported [36, 37]. Alternatively, exaggerated secretion of chylomicron particles in MetS may be indicative of intestinal hypertrophy, however this cannot be determined in this cross sectional study.

In subjects without MetS, there was a relatively weak correlation of triglyceride with apo B-48, contrasting the result observed in the MetS group. Since triglyceride concentration in plasma is mainly contributed by the triglyceride associated with VLDL particles, the association between apo B-48 and triglyceride concentration suggests a common metabolic defect that is more pronounced in those with MetS, leading to accumulation of these triglyceride-rich lipoproteins. In order to standardise for the magnitude of these parameters we also examined the association between the Z scores of apo B-48 and anthropometric and lipid/metabolic determinants. This allowed us to compare the slope of these relationships when standardised for one standard deviation change in each parameter, and also to correct for age and gender. However the slope of the association of Z-scores for most of these parameters was remarkably low, and although triglyceride had the highest slope for association with apo B-48 in the no MetS group, it had a low coefficient of variation suggesting that triglyceride is a poor surrogate marker of chylomicron remnant homeostasis. In subjects with MetS, the slope of the association of Z scores for apo B-48 with triglyceride and non-HDL cholesterol and the coefficient of variation were increased, nonetheless these measures were still not predictive of chylomicron remnant homeostasis.

In subjects with MetS the elevated triglyceride concentration is suggestive of a lipolytic defect. Although not directly measured in this study, subjects with MetS may have decreased expression of LPL as a consequence of insulin resistance [24]. A defect in lipolysis would be expected to promote an increased residency time for TRLs including chylomicron particles and favour increased lipid exchange promote accumulation of TRL remnants [38, 39]. In the plasma compartment, remodelling of lipoproteins involves the exchange of cholesteryl ester and triglyceride between triglyceride-rich (chylomicron and hepatic derived) lipoproteins and cholesterol-rich lipoproteins (LDL and HDL particles). Although we have observed an increased circulating concentration of chylomicron remnants in subjects with MetS this change was not associated with a decrease in HDL cholesterol concentrations. It seems that other factors independent to chylomicron metabolism such as HDL particle instability due to insulin resistance may implicate the low concentration of HDL cholesterol in MetS [40].

Clearance of chylomicron remnants post hydrolysis is a highly efficient process that occurs principally via internalization by LDL receptor and LDL receptor-related protein 1 after remnants were bound to the HSPG of the surface of hepatocytes facilitated by apo E [41]. Insulin resistance has been reported to associate with diminished remnant clearance by impairing the HSPG structure via modulation of hepatic SULF2 expression [42] and by altering LDL receptor via PCSK9 [43]. If accumulation of chylomicron remnants in MetS occurs principally as a consequence of impaired lipoprotein binding and/or depressed receptor expression, then by extension one would anticipate a strong association with LDL cholesterol. The findings show that in subjects with MetS, the association of LDL cholesterol with apo B-48 was strengthened in comparison to control subjects with presumably adequate expression of apo B/E receptor. The findings suggest that decreased expression may have contributed to an accumulation of chylomicron remnants in MetS subjects. However as only one-third of the variability in apo B-48 can be explained by LDL cholesterol, this does not support the contention that LDL cholesterol serves as a good surrogate marker of pro-atherogenic remnant homeostasis in plasma, and that MetS is not associated with a major defect in high affinity clearance pathways. The latter may be indicative of the findings that chylomicron remnants require greater clusters for apo B/E receptors on the plasma membrane for internalization, whereas LDL particles interact with singular receptors. A modest reduction in receptor expression will therefore have a greater effect on clearance of chylomicron remnants in comparison to LDL particles. By extension, LDL cholesterol would be a poor predictor of remnant homeostasis, particularly if receptor expression is attenuated.

Non HDL cholesterol is defined as the cholesterol associated with LDL, VLDL remnants, Lp(a), and chylomicron remnants and as these represent all the lipoproteins currently believed to contribute to atheroma development [44] this measure has been suggested to be included in routine lipid profiles. In the present study, in addition to triglyceride, non-HDL cholesterol concentration was also the major lipid contributor to the variation in apo B-48 concentration in subjects with MetS as shown by the regression analysis. Our observation of a strong association between concentrations of apo B-48 and non-HDL cholesterol in subjects with MetS is indicative of the importance chylomicron remnants to the variability of this measure, however nonetheless, as with triglyceride concentrations, there still remains a significant proportion of individuals who exhibit increased chylomicronemia in the absence of elevated triglyceride or non-HDL cholesterol concentrations. Furthermore the concentration of HDL cholesterol was not associated with apo B-48 in either group after adjustment for age and gender. These findings clearly demonstrate that HDL cholesterol is not a suitable surrogate to consider chylomicron remnant homeostasis in Caucasian subjects with MetS.

Substantial evidence has indicated that an altered chylomicron metabolism might relate to insulin resistance [45, 46]. Our results show no significant associations between apo B-48 and either insulin concentrations or HOMA-IR score, however after adjustment for age and gender there was a moderate association between insulin and concentrations, but only in the MetS group. Interestingly Kinoshita et al. [18] reported a strong association between apo B-48 and HOMA-IR score in both males and females. However differences in the ethnicity and subject characteristics may explain this discrepancy. Interestingly we found that in individuals with MetS, the association between apo B-48 concentration and some anthropometric measures was inversely correlated. This could be due to the diminished LPL activity in adipose tissue caused by insulin resistance [47]. It seems that apo B-48 concentration in circulation is associated with the inability to store fat in adipose tissue in individual with MetS. However this needs to be clarified in a larger sample size.

The results from this study have extended the findings from previous studies [18, 21, 22] that have reported the association between apo B-48 and a range of lipid and anthropometric measures in Japanese population. Our observation that apo B-48 concentration correlated with triglyceride and non-HDL cholesterol in subjects with MetS may partly explain the increased atherogenic risk in MetS, however the importance of an increased concentration of apo B-48 for atherogenic risk in this group remains. The present study has several limitations. Firstly, the cross-sectional nature of the design limits the causative interpretation between variables. Second, the older age range of our subjects may not represent the variation of apo B-48 level in general population. Third, the relatively small number of subjects with MetS may overestimate the association.

## Conclusion

In summary, our investigation confirms previous reports that fasting apo B-48 concentration is strongly associated with triglyceride concentration. Although we observed that this association was stronger in subjects with MetS there still remains a significant proportion of the variability in apo B-48 that is not predicted by components of MetS. These observations are suggestive of the importance of assessing TRL remnants, in particular chylomicron remnants in subjects with MetS. These findings suggest that subjects with MetS exhibit a lipoprotein metabolic defect the manifestation of which affects the metabolism and clearance of triglyceride-rich lipoproteins likely contributing to increased cardiovascular risk.

## Abbreviations

Apo: Apolipoprotein
BMI: Body mass index
CETP: Cholesteryl ester transfer protein
CRP: C-reactive protein
CVD: Cardiovascular disease
HDL: High density lipoprotein
HL: Hepatic lipase
HOMA-IR: Homeostasis model of insulin resistance
HSPG: Heparan sulphate proteoglycan
LDL: Low density lipoprotein
Lp(a): Lipoprotein(a)
LPL: Lipoprotein lipase
MetS: Metabolic syndrome
MTP: Microsomal transfer protein
NPC1L1: Niemann-Pick C1-like 1
PCSK9: Proprotein convertase subtilisin/kexin type 9
SAD: Sagittal abdominal diameter
SE: Standard error
SULF2: Sulfate glucosamine 6-O-endosulfatase-2
TRL: Triglyceride-rich lipoprotein
VLDL: Very low density lipoprotein
WHR: Waist hip ratio
